# Influence of Al_2_O_3_ Nanoparticle Addition on a UV Cured Polyacrylate for 3D Inkjet Printing

**DOI:** 10.3390/polym11040633

**Published:** 2019-04-06

**Authors:** Dennis Graf, Sven Burchard, Julian Crespo, Christof Megnin, Sebastian Gutsch, Margit Zacharias, Thomas Hanemann

**Affiliations:** 1 Institute for Applied Materials, Karlsruhe Institute of Technology, 76344 Eggenstein-Leopoldshafen, Germany; thomas.hanemann@kit.edu; 2 Laboratory for Materials Processing, University of Freiburg, 79110 Freiburg, Germany; sven.burchard@mars.uni-freiburg.de; 3 TECNAN, Tecnología Navarra de Nanoproductos S.L, Industrial Area Perguita, C/A No. 1, 31210 Los Arcos, Spain; julian.crespo@tecnan-nanomat.es; 4 Institute of Microstructure Technology, Karlsruhe Institute of Technology, 76344 Eggenstein-Leopoldshafen, Germany; christof.megnin@memetis.com; 5 Laboratory for Nanotechnology, University of Freiburg, 79110 Freiburg, Germany; sebastian.gutsch@imtek.de (S.G.); margit.zacharias@imtek.uni-freiburg.de (M.Z.)

**Keywords:** nanocomposites, nanoparticles, 3D Inkjet printing, ceramic inks

## Abstract

The brittleness of acrylic photopolymers, frequently used in 3D Inkjet printing, limits their utilization in structural applications. In this study, a process was developed for the production and characterization of an alumina-enhanced nanocomposite with improved mechanical properties for Inkjet printing. Ceramic nanoparticles with an average primary particle size (APPS) of 16 nm and 31 nm, which was assessed via high-resolution scanning electron microscopy (HRSEM), were functionalized with 3.43 and 5.59 mg/m^2^ 3-(trimethoxysilyl)propyl methacrylate (MPS), respectively, while being ground in a ball mill. The suspensions of the modified fillers in a newly formulated acrylic mixture showed viscosities of 14 and 7 mPa∙s at the printing temperature of 60 °C. Ink-jetting tests were conducted successfully without clogging the printing nozzles. Tensile tests of casted specimens showed an improvement of the tensile strength and elongation at break in composites filled with 31 nm by 10.7% and 74.9%, respectively, relative to the unfilled polymer.

## 1. Introduction

Three-dimensional Inkjet printing, or alternatively material jetting, is an additive manufacturing technology, which allows the fabrication of structures in a layer-by-layer fashion with a high degree of complexity. Thereby, a liquid “ink” is jetted dropwise from a print head onto a build plate and solidifies via phase change, chemical reaction, or solvent evaporation. An established method for material deposition is drop on demand (DoD) via piezo electric actuation, which enables high accuracies with a resolution of up to 34 µm [1]. It requires the material to have an optimal viscosity between 10 and 100 mPa∙s and to be compatible with small orifice diameters of 30 to 50 µm [1,2]. This technology allows the printing of several materials at once due to the presence of multiple nozzles per print head. The scope of commercial materials allows the production of components with varying degrees of hardness, flexibility, electrical properties, and biocompatibility [3]. Often these materials are UV curable photopolymers, which have the advantage of instant chemical solidification and low emission [2,4]. However, in the area of structural applications, for example in dentistry [5], where high stiffness and toughness are required, photopolymers show their disadvantages. Most of the available materials are highly cross-linkable thermosets such as (meth)acrylates and epoxies and are therefore rather brittle [5,6]. In the past two decades, nanomaterials, especially nanoparticles, were extensively investigated as fillers in polymer matrix composites [7,8,9]. The addition of even small amounts of the nano phase showed to increase the Young’s modulus of the composite without compromising the toughness [10,11,12]. In thermosets, the investigation of the “nano” effect highlighted several mechanisms as responsible for the improvement. Among these are crack deflection, particle pull-out, microcracking, and plastic void growth. The latter is regarded as the most dominant mechanism to influence the toughening [8,13,14]. It involves the debonding of the nanoparticle from the matrix and a plastic growth of a void around the filler. Although only little energy is absorbed when compared with the plastic deformation of the matrix, the mechanism causes constraint reduction of the crack tip during formation. This mechanism depends on the size and distribution of the fillers, whereby homogeneous particle dispersions with filler diameters below 50 nm are expected to show significant improvements [14]. Furthermore, the surface treatment, which defines whether the interface is weak or strong, is of importance, and examples for effective toughening have been shown for both cases [13,15]. However, the challenge in utilizing nanomaterials is their increased tendency to form agglomerates and the high effort to separate them [16]. Commercial nanoparticles in their as-received form often exhibit an aggregated structure, in which the primary nanoparticles are connected by hard bridges. Additionally, the aggregates themselves are interlinked to superordinate structures of hard agglomerates [17]. To harness the full advantage of the strengthening phase, strong shear forces have to be utilized to break it apart. Furthermore, sterically stabilizing ligands have to be attached to prevent re-agglomeration. This is most often done with 3-(trimethoxysilyl)propyl methacrylate (MPS), which attaches in a condensation reaction to the surface of the nanoparticles, after hydrolysis of its alkoxy groups [18]. Due to the presence of surface bound water, a selective reaction with the ceramic can be achieved [15]. In spite of the attached molecules, nanoparticles tend to agglomerate in matrix anyway so that an optimal nanoparticle concentration is often observed, after which a decline of the improved mechanical properties occurs [19,20]. In addition to stabilization, silanes like MPS are utilized as co-photoinitiators, which have to be considered when the polymerization parameters are chosen [21,22]. 

In this work, a manufacturing and characterization process for a 3D Inkjet printable nanocomposite was developed. First, high-resolution scanning electron microscopy (HRSEM) images of the fillers were made to measure the primary particle size distribution and to assess the extent of particle aggregation. Second, mechanical grinding and functionalization with the ligand MPS was done to decrease the size of hard agglomerates and increase the compatibility of the particles with the organic matrix. The functionalized fillers were characterized via thermogravimetric analysis (TGA) and Fourier-transform infrared (FTIR) spectroscopy. Third, a UV-curable ceramic ink was formulated by suspending the functionalized fillers in a photoinitiator-containing mixture of mono- and polyfunctional acrylates. The dispersion was characterized by determining the particle sized distribution (PSD) of the fillers in the matrix, measuring the dynamic viscosity and conducting ink-jetting tests. After preparation of specimens by casting, tensile tests were conducted. The goal of the process was the fabrication of a new material for 3D Inkjet printing, which utilizes toughening mechanisms initiated by ceramic nanoparticle in order to increase the tensile strength and elongation at break of brittle acrylic photopolymers. According to our knowledge, investigation in this area of materials for Inkjet printing are very limited and rather confined to the related field of stereolithography [23,24].

## 2. Materials and Methods

### 2.1. Materials

**Figure 1:**
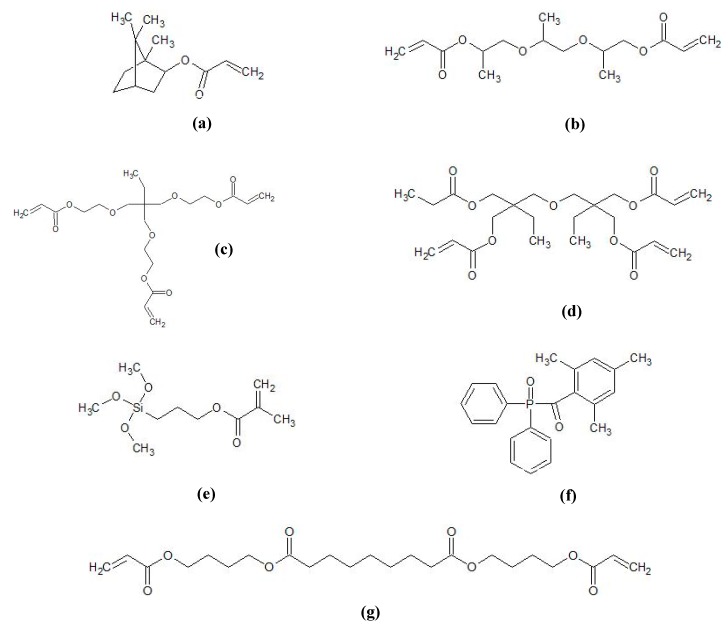
Chemical structures of the organic ink components: (**a**) Isobornyl acrylate (IBOA), (**b**) tripropylene glycol diacrylate (TPGDA), (**c**) trimethylolpropane (EO)3 triacrylate (TMPEO3TA), (**d**) di(trimethylolpropane)tetraacrylate (DTMPTA), (**e**) 3-(trimethoxysilyl)propyl methacrylate (MPS), (**f**) diphenyl(2,4,6-trimethylbenzoyl)phosphine oxide (DPO), and (**g**) polyester acrylate Genomer 3364. The precise chemical structure of the Genomer 3364 is confidential, so that the depicted molecule should act as example.

Aluminum oxide nanoparticles with an average particle size (APS) of 16 nm (TEC13Al_2_O_3_ TECNAN, Los Arcos, Navarre, Spain) and 31 nm (TEC35Al_2_O_3_ TECNAN, Los Arcos, Navarre, Spain) were used as received. The BET gas adsorption showed a specific surface area of 126 m^2^/g and 43 m^2^/g, respectively. Isobornyl acrylate (IBOA, Rahn Chemicals, Zurich, Switzerland), tripropylene glycol diacrylate (TPGDA, Arkema, Colombes, France), trimethylolpropane (EO)3 triacrylate (TMPEO3TA, KPX green chemicals, Seosan, South Korea), di(trimethylolpropane)tetraacrylate (DTMPTA, Sigma Aldrich, Darmstadt, Germany), Genomer 3364 (Rahn Chemicals, Zurich, Switzerland), 3-(trimethoxysilyl)propyl methacrylate (MPS, Sigma Aldrich, Darmstadt, Germany), ethylene glycol dimethacrylate (EGDMA, Merck, Darmstadt, Germany), potassium bromide (KBr, Sigma Aldrich, Darmstadt, Germany), and the photoinitiator diphenyl(2,4,6-trimethylbenzoyl)phosphine oxide (DPO, TCI GmbH, Eschborn, Germany) were used as received (Figure 1). 

### 2.2. Methods

#### 2.2.1. Primary Particles Size Distribution

**Figure 2:**
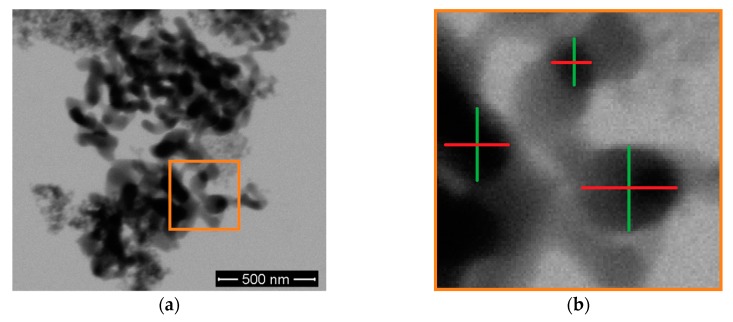
(**a**) Depiction of agglomerated aggregates. (**b**) The close-up shows that each of the primary particles were measured horizontally (red lines) and vertically (green lines) and the average was calculated.

High-resolution scanning electron microscopy (Nova NanoSEM with EDAX EDX, FEI, Hillsboro, OR, USA) images were taken using the STEM mode to determine the primary particles size distribution in the Al_2_O_3_ nanoparticles. For the preparation, each of the two kinds of as-received nanoparticles were suspended, diluted, and re-suspended in ethanol until a calculated concentration of 100 µg/mL was reached. Then, one drop of each dispersion was positioned on a carbon coated TEM grid and dried for 12 h. After the microscopy, one hundred primary nanoparticles were measured using the software ImageJ. As depicted in Figure 2, the diameter of every particle was taken horizontally (red lines) and vertically (green lines), the average of the two values was calculated and the frequency of the nanoparticle was plotted against the diameter.

#### 2.2.2. Grafting MPS onto Al_2_O_3_ Nanoparticles

The silane was grafted onto Al_2_O_3_ particles while being ground in a ball mill (PBM, PM400, Retsch GmbH, Haan, Germany). For the functionalization of the 16 nm sized fillers, with a specific surface area (SSA) of 126 m^2^/g, 7.1 wt % of the as-received particles, 3.5 of MPS, 29.2 wt % of ethanol, and 60.2 wt % of ZrO_2_ grinding balls, with a diameter of 2 mm, were filled into two 125 mL grinding jars with a ZrO_2_ interior cladding. The content was treated for 4 h at 200 rpm. Afterwards, the suspension was dried in a rotary evaporator for 1 h at 175 mbar and 50 °C. Then 100 mg of the dried powder was characterized via thermogravimetric analysis (STA 409C, Netsch GmbH & Co. KG, Selb, Germany) in air atmosphere up to 900 °C at a heating rate of 10 K/min. Fourier transform infrared spectroscopy (Excalibur series, Bio Rad Laboratories, Inc., Hercules, California, USA) was done in rapid scan mode between 750 and 4000 cm^−1^ with a sensitivity of 16. For each sample, 32 scans were conducted under a constant nitrogen flow of 11 l/min. The samples were prepared by pressing a mixture of 200 mg of KBr and 2 mg of the functionalized powder into pellets using a hydraulic press (Specac Ltd, Orpington, United Kingdome) with a compressive load of 10 t for 10 min. The background measurement was conducted with a pure KBr pellet. 

The 31 nm sized powder, with a SSA of 43 m^2^/g, was treated and characterized using the same procedures. The content of the grinding jars during grafting was 8.6 wt % particles, 1.3 wt % MPS, 17.0 wt % ethanol and 73.1 wt % ZrO_2_ grinding balls. Based on the TGA results, the amount of grafted MPS relative to the particle surface area was calculated using,

(1) $$wt\%\left(MPS\right)=wt\%\left(loss\right)\cdot \frac{M\left(MPS\right)}{M\left(MPS_{org}\right)\cdot 100}\cdot 100$$

with wt % (MPS) being the weight percentage of grafted molecules on the nanoparticles, wt % (loss) the maximum weight loss of a sample in the TGA, M (MPS) the molar mass of MPS, and M (MPS_org_) the molar mass of the organic components of MPS. 

#### 2.2.3. Preparation and Characterization of Ceramic Inks

**Figure 3:**
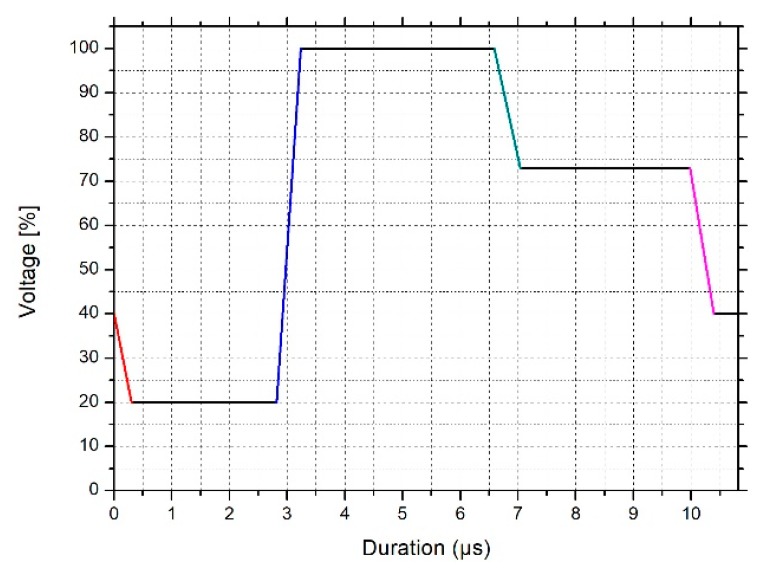
Waveform of the DMC-11610 printhead adjusted for jetting tests with the ceramic inks.

The functionalized powders, MPS-TEC13Al_2_O_3_ and MPS-TEC35Al_2_O_3_, were re-dispersed in an acrylate matrix using a T 10 basic Ultra-Turrax (IKA, Staufen, Germany) at 14450 rpm for 5 min. The matrix comprised 45.7 wt % IBOA, 26.5 wt % TPGDA, 10.9 wt % TMPEO3TA, 3.5 wt % DTMPTA, 12.0 wt % Genomer 3364, and 1.4 wt % DPO. Suspensions with different ceramic contents were produced, shown in table 1. The PSD of the dispersions was investigated with a NANO-flex® (Particle Metrix GmbH, Inning, Germany). For the measurement, 160 µL of the suspensions was dropped into 5 g of EGDMA each. The recording of the background signal was done using high purity water. Each sample was measured three times, whereby the sample was retrieved and vigorously shaken between the recordings. The dynamic viscosity was investigated with an automated dynamic shear rheometer CVO 50 (Malvern Instruments, Malvern, UK) at a constant shear rate of 200 s^−1^ at a temperature between 20 °C and 80 °C. For the measurement, a cone-plate setup was chosen with a cone diameter of 60 mm and an angle of inclination of 2°. Jetting tests were conducted with a Dimatix Materials Printer DMP-2850 (Fujifilm Dimatix, Inc., Santa Clara, CA, USA) using the drop view. DMC-11610 cartridges were used with a drop volume of about 10 picoliters and a print head temperature of 60 °C. The firing voltage was set to 30 V and the waveform, which controls the bending of the piezo-electric actor in the printhead and influences the drop formation, was adjusted for the ceramic inks (Figure 3). The waveform is subdivided into four phases. In the transition from the standby position at 40% of the firing voltage into the first phase at 20% of the firing voltage, the slew rate, which is seen as the red slopes in the waveform, is 0.66 and the duration is 2.816 µs. Thereby, the ceramic ink is pumped into the firing chamber by the piezo-electric membrane. In the second phase, which lasts for 3.776 µs, the ink is pushed out of the nozzle, and the curve rises to 100% at a slew rate of 1.90 (blue slope). During the third phase the curve drops from 100% to 73% at a slew rate of 0.60 (green slope) and it lasts for 3.392 µs. Finally, in the fourth phase the curve goes back to standby from 73% to 40% with a slew rate of 0.80 (pink slope) and a duration of 0.832 µs. This causes the formed drop to separate from the nozzle. 

#### 2.2.4. Tensile Testing 

Tensile tests were performed on cured samples using an Instron 5985 (Instron, Norwood, MA, USA). The samples were produced by pouring layers of pure acrylate matrix or the ceramic inks depicted in Table 1 into a polytetrafluorethylene mold. Each layer was cured with a UV light source (Dr. Hönle AG, Gräfelfing, Germany) at 405 nm and a specific power of 60 mW/cm^2^. Three specimens of each kind were produced according to the DIN EN ISO 527-2 type A1 standard. The test was conducted without a pre-strain at a strain rate of 2 mm/min, whereby a 2000 N load cell was used to record the resulting stress.

## 3. Results

### 3.1. Primary Particle Size Distribution

**Figure 4:**
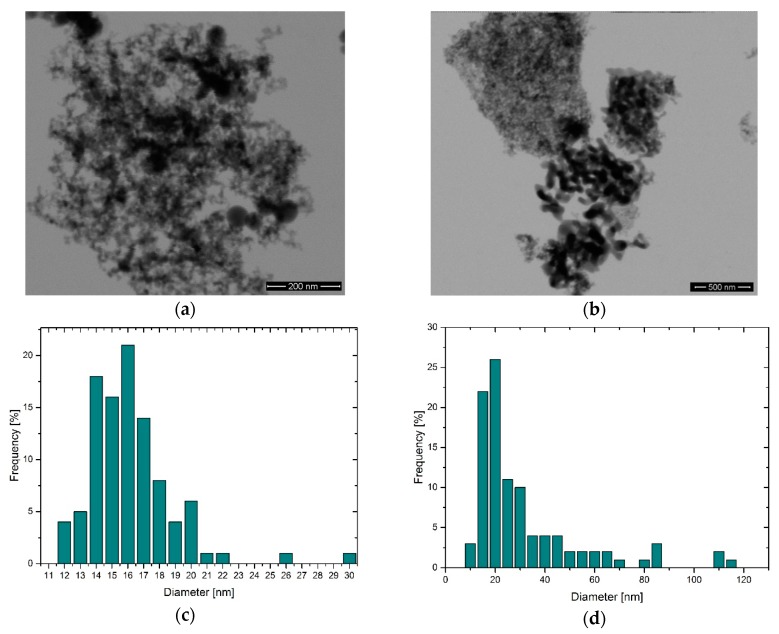
Results of the high-resolution scanning microscopy (HRSEM) analysis of the powders TEC13Al_2_O_3_ (**a**) and TEC35Al_2_O_3_ (**b**). Results of particle size counting using the images showed a narrow particle size distribution (PSD) for the former (**c**) and a broader PSD for the latter (**d**) powder.

HRSEM analysis showed that TEC13Al_2_O_3_ exhibits a narrow primary particle size distribution (PPSD) with an APS of 16 nm, a D10 of 13 nm, a D50 of 15 nm, and a D90 of 19 nm (Figure 4a,c). For TEC35Al_2_O_3_, the PPSD was 31 nm (Figure 4b,d). The particles were more heterogeneous with a D10 of 12 nm, a D50 of 20 nm, and a D90 of 60 nm. In the images, necks between the primary nanoparticles are visible, especially for larger primary particles, which indicate the presence of aggregates and hard agglomerates.

### 3.2. Grafting MPS on Al_2_O_3_ Nanoparticles

**Figure 5:**
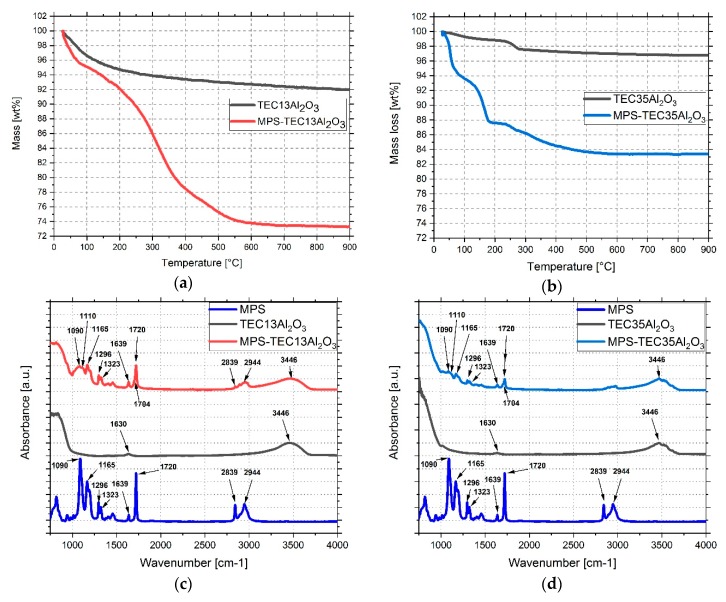
TGA curves of (**a**) as received TEC13Al_2_O_3_ and MPS-TEC13Al_2_O_3_ and (**b**) as received TEC35Al_2_O_3_ and MPS-TEC35Al_2_O_3_. Fourier-transform infrared (FTIR) spectra of MPS and the respective untreated and functionalized nanopowders of (**c**) 16 nm and (**d**) 31 nm average particle size (APS).

In presence of the particle surface bound water, MPS molecules can hydrolyze and attach chemically, in a condensation reaction, to the hydroxyl groups of the ceramic. Comparing the TGA of MPS treated and as received particles, a significantly higher weight reduction for the former is visible (Figure 5a,b). For MPS-TEC13Al_2_O_3_ it was 26.78 wt % and that of MPS-TEC35Al_2_O_3_ was 17.18 wt %. Using equation 1, the MPS loading relative to the particle surface areas is 3.43 mg/m^2^ and 5.59 mg/m^2^ respectively. The FTIR spectra (Figure 5c,d) for MPS-TEC13Al_2_O_3_ and MPS-TEC35Al_2_O_3_ were found to be equal. Compared to their non-functionalized counterparts, the presence of MPS in the FTIR results can be seen clearly. Examining the spectrum of the two untreated powders, signals at 1630 and 3446 cm^−1^ are visible, which are attributed to stretching and bending vibration modes of water adsorbed on the nanoparticles’ surface [25]. For MPS at 1165, 1296, and 1323 cm^−1^, the –C–CO–O– skeletal vibrations from the methacryloxy group are visible [26]. The peak at 1720 cm^−1^ is the carbonyl vibration of the pure unhydrolyzed molecule and at 2839 cm^−1^ as well as 2944 cm^−1^ the stretching vibrations of C–H bonds are visible. The modified Al_2_O_3_ nanoparticles have a signal at 1090 cm^−1^, which is related to the Si–O–C bonds of the MPS. It decreases after attachment onto the particles, showing that condensation has occurred. At 1110 cm^−1^ the functionalized particles exhibit the presence of Si–O–Si bonds hinting at MPS homocondensates.

The peak at 1634 cm^−1^ belongs to stretching vibrations of the C=C band, which overlaps with the water band at 1630 cm^−1^ [27,28]. At 1704 cm^−1^ the stretching vibration of the C=O group of MPS is visible, whereas 1720 cm^−1^ is a characteristic peak for carbonyl groups, which form hydrogen bonds with hydroxyl groups on the particle surface or with other silanes [27].

### 3.3. Characterization of Ceramic Inks

**Figure 6:**
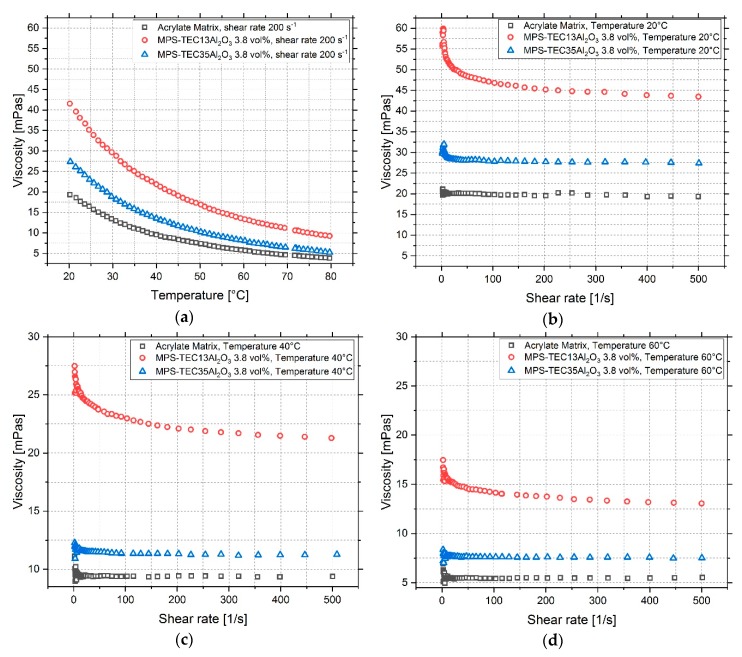
Viscosity of the unfilled, the MPS-TEC13Al_2_O_3_, and MPS-TEC35Al_2_O_3_ filled matrix (**a**) Temperature dependent decrease of the viscosity. (**b**–**d**) Non-Newtonian shear-thinning behavior of the materials at 20, 40, and 60 °C, respectively.

**Figure 7:**
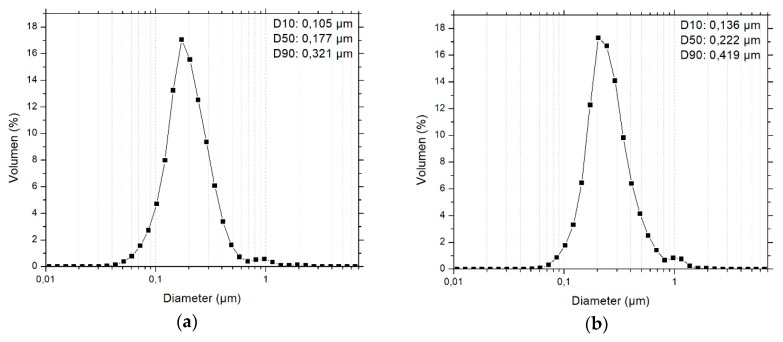

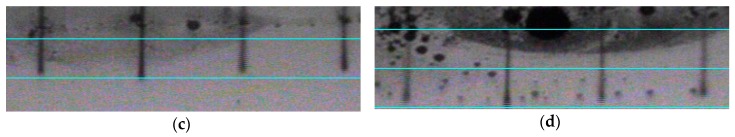
PSD of (**a**) MPS-TEC13Al_2_O_3_ and (**b**) MPS-TEC35Al_2_O_3_ in the acrylic matrix. (**c**) Droplet formation during jetting tests with the Dimatix material printer for MPS-TEC13Al_2_O_3_ in matrix (3.8 vol %) and (**d**) MPS-TEC35Al_2_O_3_ in matrix (3.8 vol %).

With increasing temperature, the viscosity reduces for all investigated materials (Figure 6a). The particle-filled systems exhibit a higher viscosity than the unfilled matrix, and MPS-TEC13Al_2_O_3_ containing inks are more viscose than those containing MPS-TEC35Al_2_O_3_. At the same time, the influence of temperature is more pronounced for the smaller particles. For the printing temperature of 60 °C, which was used for jetting tests, the viscosity was 14 and 7 mPa∙s for suspensions with 16 nm sized fillers and 31 nm sized fillers, respectively. All investigated materials showed a non-Newtonian, shear-thinning behavior with rising shear rates (Figure 6b–d). This effect is amplified during the temperature increase from 20 to 60 °C. The PSDs of the two suspensions with MPS-TEC13Al_2_O_3_ and MPS-TEC35Al_2_O_3_ show significantly larger ceramic agglomerates than the primary particle sizes (Figure 7a,b). The largest structures are smaller than 2 µm and the D90 values are 0.321 and 0.419 µm for MPS-TEC13Al_2_O_3_ and MPS-TEC35Al_2_O_3_, respectively. Ink jetting tests, show that both materials can be ejected from the nozzles without occluding them (Figure 7c,d). 

### 3.4. Tensile Tests

**Figure 8:**
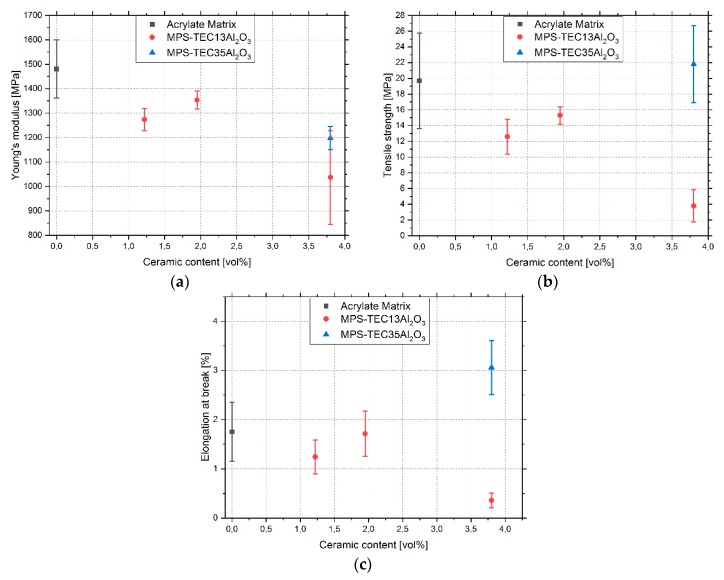
Results of the tensile test for unfilled, MPS-TEC13Al_2_O_3_, and MPS-TEC35Al_2_O_3_ filled polyacrylate matrix with (**a**) Young’s modulus, (**b**) tensile strength, and (**c**) elongation at break.

**Figure A1:**
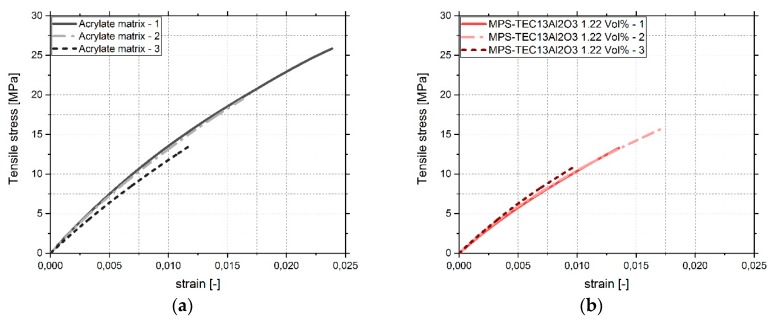

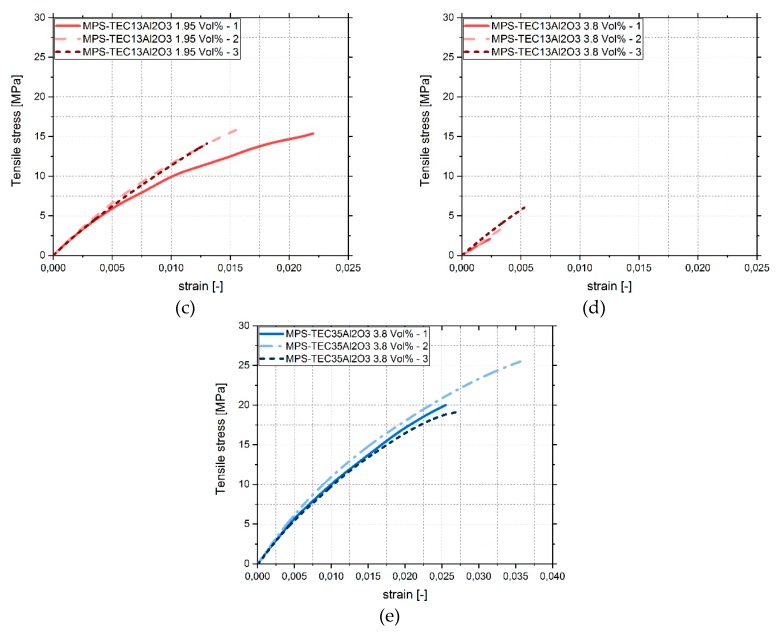
Results of the tensile test. Stress-strain curves for (**a**) unfilled, (**b**–**d**) MPS-TEC13Al_2_O_3_, and (**e**) MPS-TEC35Al_2_O_3_ filled polyacrylate matrix.

As seen in Figure 8, the values for the Young’s modulus, tensile strength, and elongation at break for the unfilled acrylate are at 1481 MPa, 19.7 MPa, and 1.75%, respectively. With introduction of ceramic nanoparticles, the Young’s modulus exhibits a drop for both variants. After a maximum at 2 vol % and a modulus of 1354 MPa, MPS-TEC13Al_2_O_3_ filled samples show a further decrease of the values with higher ceramic content. Samples with MPS-TEC35Al_2_O_3_ fillers exhibit a maximum at 1198 MPa. The tensile strength as well as the elongation at break decrease for the 16 nm ceramic-filled composite showing the best values of 15.3 MPa and 1.71%, respectively. The 31 nm ceramic-filled material improved compared to unfilled material with 21.8 MPa and 3.06% for tensile strength and elongation at break, respectively. Figure A1a–e contain the stress-strain curves, all of which show a brittle behavior. 

## 4. Discussion

Previous publications have shown that the addition of well-dispersed nanoparticles into a polymer can lead to an increase of the Young’s modulus, tensile strength, and elongation at break [13,19,29]. Purposely weakening or strengthening the interface between the filler and the matrix may even result in pronounced increase of the elongation at break [13,15]. In this work, all specimens show a brittle fracture (Figure A1) whereby significant increases in ductility could not be observed. The MPS-TEC13Al_2_O_3_ nanoparticle filled composites show their best values at 2 vol % filling content with 1354 MPa, 15.3 MPa, and 1.71% for the Young’s modulus, tensile strength, and elongation at break, respectively. This is a change of −8.6%, −22.3%, and −2.3% relative to the pure polymer. The MPS-TEC35Al_2_O_3_ nanoparticle filled material shows values of 1198 MPa, 21.8 MPa, and 3.06%, respectively. This is a change of −19.1%, 10.7%, and 74.9% relative to the pure polymer. The results show, with the exception of the specimens containing the 31 nm fillers, a general worsening of the mechanical properties. Reasons for that are on the one hand, the agglomeration of the aggregated nanoparticles with increasing ceramic content. As was previously observed [19,20,30], nanomaterial addition improves the composite properties up to a certain filling grade, after which the properties start to decline again. The same behavior can be seen for the 16 nm sized fillers, where the increase from 1.22 to 1.95 vol % leads to a local maximum of the tensile properties to shrink again for 3.8 vol %. On the other hand, the ligand MPS contributed not only to the stability of the nanoparticles in the organic matrix but, as a silane, it is known to act as a co-photoinitiator [21,31]. According to literature, about 1.49 mg/m^2^ is needed to form a closed organic shell [32,33,34,35,36]. The increased molecular loading in the results of 3.43 and 5.59 mg/m^2^ for MPS-TEC13Al_2_O_3_ and MPS-TEC35Al_2_O_3_ respectively can be explained by physical MPS adsorption in addition to covalent binding, as was shown in the FTIR spectra. An excess of the molecule may cause a shortening of the polymer chain length and reduce the number of crosslinks [22,37]. This may result in a reduction of the stiffness and a further increase in brittleness, which is especially true for the 16 nm particle filled composites due to a larger amount of attached MPS [38,39]. The reduced 5.59 mg/m^2^ on the 31 nm fillers in combination with the opaqueness of the composite, caused by the light scattering of aggregates, might have attenuated the adverse effects of the silane and contributed to the higher strength and elongation at break relative to other tensile samples [2]. The reason for the enhancement in the MPS-TEC35Al_2_O_3_ filled samples is potentially the particle void growth mechanism as described above, whereby the debonding of the nanofillers leads to energy dissipation at the tips of propagating cracks [13,14]. 

Consequently, it is possible to formulate 3D ink-jettable nanocomposites with adjusted mechanical properties using current commercial materials. For the fabrication process, a time saving one pot ceramic grinding and MPS grafting step was utilized, which allows a large-scale functionalization with a subsequent drying step of the nanoparticles. The treatment in the ball mill enables the break up of hard agglomerates between the aggregated nanopowder which can be seen in Figure 4a,b. The size reduction of the agglomerates is necessary in order to increase the ceramic matrix interface which contributes to the toughening of the composite [17]. The grafting of the silane molecules onto the particles was catalyzed by the surface-bound water, which enables a localized hydrolysis and condensation of the molecules and prevented unwanted crosslinking of the coupling agent [15]. The newly formulated acrylic mixture has suitable rheological properties for Inkjet printing (Figure 6a–d), with a viscosity of 5 mPa∙s at 60 °C and allows the UV curing in an ambient atmosphere. The re-dispersion of MPS-grafted nanoparticles in the organic matrix yielded stable suspensions with a non-Newtonian behavior. The viscosity of the dispersions and of the unfilled acrylate matrix is influenced by the molecular interactions within the matrix, by particle-particle interactions, and by matrix-particle interactions (Figure 6) [40]. The particle-filled systems exhibit a higher viscosity than the unfilled matrix due to the flow hindering effect of the fillers. The acrylate surrounding the nanoparticles additionally adheres to their surface, further increasing the viscosity [17,41]. At the printing temperature of 60 °C values of 14 and 7 mPa∙s for the 16 nm and the 31 nm particle filled ink, respectively, were observed. The D90 for MPS-TEC13Al_2_O_3_ and MPS-TEC35Al_2_O_3_ filled materials was 0.321 and 0.419 µm, respectively, which again underlines the presence of agglomerated and aggregated particles. Nevertheless, occlusion of the printing nozzles was not observed during the jetting trials. However, higher ceramic concentration beyond 3.8 vol % might bear the risk of increased particle agglomeration [2].

## 5. Conclusions

The study has introduced a UV curable and 3D ink-jettable acrylic material filled with MPS stabilized Al_2_O_3_ nanoparticles with an average primary particle size of 16 nm and 31 nm for structural applications. For the fabrication of the material, commercial ceramics were ground and functionalized in a ball mill. It was shown that MPS attached chemically and physically to the nanoparticle surface. The dispersion of the stabilized fillers in a newly formulated low viscose matrix resulted in suspensions with suitable rheological properties and PSDs for Inkjet printing. Tests with a commercial printer exhibited no occlusion of nozzles during jetting. For MPS-TEC35Al_2_O_3_ filled tensile specimens with a filling grade of 3.8 vol %, the test results showed an improvement of up to 10.7%, and 74.9% for the tensile strength and elongation at break, respectively, relative to the pure polymerized matrix. The other composites showed no improvement relative to the unfilled polymer. The results indicate that the incorporation of nanofillers into a brittle photoresin can be a viable way of increasing the mechanical properties and might contribute to the formulation of a 3D Inkjet printable structural material.

## Figures and Tables

**Table 1:** Overview of the filler content of the prepared ceramic inks.

Filler	Content [vol %]
MPS-TEC13Al_2_O_3_	1.22
MPS-TEC13Al_2_O_3_	1.95
MPS-TEC13Al_2_O_3_	3.80
MPS-TEC35Al_2_O_3_	3.80
